# The National Pension Fund

**Published:** 1887-06-25

**Authors:** 


### THE NATIONAL PENSION FUND.

Many of our fair correspondents continue to echo an old refrain. They say: "We cannot send you our names for your Pension Fund until we know the conditions." Most wise and cautious ladies; but, as we have explained already, we do not ask you to send in your names for *our* or *any particular Pension Fund.* What we do ask is this—Are you willing to subscribe to a Pension Fund if the conditions suit you when they appear? Are you willing to marry? That is one question. Are you willing to marry Mr. Smith? That, you perceive, is quite another. We do not ask you now to commit yourself to Mr. Smith, but simply to tell us whether or not you will marry if Mr. Eligible should ask you at same future time. Sending in your names commits you to nothing. There are nearly three hundred now sent in. When this number is increased to two thousand the Fund can be definitely started.

